# Reliability of monitoring acid‐base and electrolyte parameters through circuit lines during regional citrate anticoagulation‐continuous renal replacement therapy

**DOI:** 10.1111/nicc.12696

**Published:** 2021-08-11

**Authors:** Fang Wang, Mingjin Dai, Yuliang Zhao, Yingying Yang, Zhiwen Chen, Li Lin, Xue Tang, Ling Zhang

**Affiliations:** ^1^ Department of Nephrology, West China Hospital, Sichuan University/West China School of Nursing Sichuan University Chengdu Sichuan China; ^2^ Deparment of Nephrology, West China Hospital Sichuan University Chengdu Sichuan China

**Keywords:** access recirculation, acid‐base and electrolytes, continuous renal replacement therapy, regular connection, reversed connection

## Abstract

**Background:**

The current practice involves blood sampling from the circuit line to measure acid‐base and electrolyte parameters during continuous renal replacement therapy (CRRT). However, there is limited evidence supporting its reliability due to the effects of anticoagulant mechanism and access recirculation associated with regional citrate anticoagulation (RCA).

**Aim:**

To evaluate the reliability of monitoring acid‐base and electrolyte parameters through circuit lines in regular and reversed connections during RCA‐CRRT.

**Study design:**

In this prospective cohort study, we included critically ill patients receiving RCA‐CRRT via a double‐lumen catheter. During the second hour after CRRT initiation, we collected blood samples to monitor acid‐base and electrolyte parameters and their levels were compared between samples from the circuit lines (at 0, 3, and 5 minutes) and those from the central venous catheter (CVC) line (at 0 minute). During this time, CRRT switched to the replacement state as controls.

**Results:**

We observed 128 CRRT circuits in 60 adult patients receiving RCA‐CRRT. Ninety‐eight (76.6%) circuits had regular connections, while 30 (23.4%) had reversed connections. Among regular connections, no differences were observed in any acid‐base or electrolyte parameters between samples from the CVC line and those from the circuit line at all time points (*P* > .05). Among reversed connections, ionized calcium levels were dramatically decreased at all three time points in samples from the circuit line compared with those from the CVC line (0.65 ± 0.12, 0.72 ± 0.11, and 0.78 ± 0.99 vs 0.98 ± 0.07 mmol/L, *P* < .001), with comparable levels of other acid‐base or electrolyte parameters between the sampling patterns (*P* > .05).

**Conclusions:**

Acid‐base and electrolyte parameters could be reliably monitored through the circuit line during RCA‐CRRT in regular connections. However, in reversed connections, pre‐filter ionized calcium concentrations determined through the circuit line were lower than those determined through the CVC line.

**Relevance to clinical practice:**

We suggest sampling from arterial or CVC lines rather than from the circuit line in a reversed connection during RCA‐CRRT.

**What is known about the topic**

- Many continuous renal replacement therapy (CRRT) circuits have reversed blood lines to overcome catheter dysfunction.
- The access recirculation rate is higher with a reversed connection.
- Blood sampling from the circuit line during CRRT with regional citrate anticoagulation (RCA) is technically possible, but it raises a question regarding the effect of access recirculation on the accuracy of the measured parameters during CRRT with RCA.
- Currently, there is a lack of evidence regarding the best method for blood sampling through the circuit line during CRRT with RCA.

**What this paper adds**

- Acid‐base and electrolyte parameters can be reliably monitored from the circuit line in a regular connection during CRRT with RCA.
- There is no need to stop CRRT while collecting blood samples through the circuit line in a regular connection during CRRT with RCA.
- It is inaccurate to monitor pre‐filter ionized calcium concentration through the circuit line when a reversed connection is used during CRRT with RCA.

## 1 INTRODUCTION

Continuous renal replacement therapy (CRRT) is one of the most important methods for maintaining electrolyte and acid‐base homeostasis in critically ill patients with or without acute kidney injury.^1^ Systemic anticoagulation is often required to prevent clotting of the filter and extracorporeal circulation. Compared with heparin, regional citrate anticoagulation (RCA) is safer and confers a longer circuit life and consequently less circuit downtime.^2^, ^3^, ^4^ Citrate is commonly recommended as the first‐line anticoagulant for CRRT in critically ill patients. It acts as an anticoagulant by chelating ionized calcium in the extracorporeal circuit.^5^, ^6^ CRRT using RCA as an anticoagulation protocol usually requires a complex monitoring system due to continuous adjustment of calcium infusion and citrate flow to achieve an ideal pre‐filter and post‐filter ionized calcium levels.^7^, ^8^ Reportedly, electrolyte disturbances are among the most common adverse events observed immediately after the initiation of CRRT with RCA.^9^, ^10^ It is critical to perform individual adjustments in the CRRT parameters based on the results of the blood sample analysis.^11^ Therefore, an accurate test for blood samples is essential to evaluate electrolytes during CRRT with RCA in critically ill patients.

Intravenous double‐lumen catheters are commonly used for temporary vascular access in CRRT. The clinical complications associated with temporary central venous catheters (CVCs) are attributable to a variety of reasons, including thrombus formation, fibrin sheath formation, and malposition.^12^ A therapeutic approach commonly applied in daily clinical practice to overcome the barrier of catheter dysfunction, to attain sufficient blood flow rates, and to provide adequate dialysis to lengthen the duration of renal replacement therapy involves reversing the connection mode of dysfunctional double‐lumen CVCs,^12^ which consists of an arterial line and a venous line. However, access recirculation increases with both temporary and permanent functional catheters after reversing the arterial and venous lines.^13^ The reported rate of access recirculation is less than 5% with a correct connection, but increases to 13% with a reversed connection.^13^, ^14^, ^15^, ^16^, ^17^ Increased access recirculation due to reversed lines may impair dialysis adequacy and test accuracy of blood samples from the circuit.

Although arterial blood gas analysis is considered the gold standard for measuring acid‐base balance and electrolyte parameters, some CRRT patients have no peripheral arterial line in the clinical scenarios, such as dislodged the arterial lines, difficult access, and patients with stable cardiovascular who require long‐term CRRT. Therefore, sampling from circuit lines is the standard practice for measuring acid‐base and electrolyte parameters in some countries. Every CRRT extracorporeal circulation line, which consists of an arterial line and a venous line, has an arterial sampling port, which makes the system simple and convenient for blood sampling. However, ionized calcium could be chelated by citrate anticoagulation when blood is sampled from the circuit line during CRRT with RCA and the reliability of monitoring electrolytes, particularly ionized calcium, has rarely been discussed with respect to access recirculation. To the best of our knowledge, only two case reports have indicated that blood sampling from the circuit line during CRRT with RCA could not avoid the effects of vascular access recirculation on the determination of ionized calcium levels.^18^, ^19^ Therefore, we raised the question of the accuracy of blood sampling from the circuit line during CRRT with RCA, especially in a reversed connection.

The aim of this study was to evaluate the accuracy and timing of monitoring the acid‐base and electrolyte parameters through the CRRT circuit line by comparing them with the blood sampling results from the CVC line during RCA‐CRRT.

## 2 MATERIALS AND METHODS

### 2.1 Design, setting, and sample

The present study was a protocol‐designed, prospective, cohort study requiring blood sampling at set time places (CVC line and circuit line) and time points (at 0, 3, and 5 minutes during the second hour after CRRT initiation) during the replacement state with only the blood pump running. Patients who underwent CRRT were recruited from the intensive care unit of West China Hospital from December 2019 to June 2020.

Inclusion criteria were patients requiring CRRT; indication for RCA; patients with haemodynamic stability; current use of temporary CVCs without an arterial line in situ; patients aged 18 to 70 years old; and patients who provided informed consent.

Exclusion criteria were patients who underwent blood transfusion within the last 24 hours; patients who required blood products during the CRRT session; and patients with decompensated liver disease haemostatic disorders, coagulation derangements, allergies to citrate products, and active CVC‐related bloodstream infection. The progress of circuits in this study is summarized in Figure S1.

### 2.2 Ethical approval

This study was approved by the Research Ethics Committee (Approval No. 2019‐1048). Informed consent was obtained from all patients and/or their guardians before the commencement of CRRT and any sampling procedures.

### 2.3 Study Protocol

Double‐lumen catheters (13Fr, 250 mm; GDHK‐1325, Baxter International Inc., Deerfield, Illinois) were placed in the femoral veins. Post‐dilution continuous veno‐venous haemodiafiltration (CVVHDF) was performed using the Prismaflex machine and ST150 haemofilters (Baxter International Inc.), anticoagulation module was selected systemic anticoagulation in the Prismaflex machine, which facilitated the adjustment of citrate parameters in our hospital. CRRT was set at 25 to 35 mL/kg/h (dialysis: replacement fluid = 1:1), a commercial calcium‐containing solution (Ca^2+^, 1.6 mmol/L) (Qingshan Likang, Pharmaceutical Co., Ltd., Chengdu, China) was used as the dialysis and replacement solution. All patients received regional anticoagulation with 4% trisodium citrate solution (target post‐filter ionized calcium level: 0.25‐0.35 mmol/L) and 10% calcium gluconate was administered on an as‐needed basis as an intermittent intravenous bolus to maintain the plasma ionized calcium level >0.9 mmol/L. The blood flow rate was 130 to 150 mL/min during RCA‐CRRT. All samples for monitoring the acid‐base and electrolyte parameters were collected from both the regular CVC line and the arterial sampling point of the circuit line with patients in the supine position. The CVC line was not used for CRRT. The blood sample collection for measuring the acid‐base and electrolyte parameters was based on a fixed time course, starting from the second hour of CRRT (set as 0 minute). We collected blood samples from the circuit line at 0 minute with CRRT in the CVVHDF state and at 3 and 5 minutes as controls. During this time, CRRT was switched to the replacement state with only the blood pump running to decrease the citrate concentration of the circuit. Blood samples were collected from the CVC line at 0 minute. The circuit with the arterial sampling point of RCA‐based CVVHDF is shown in Figure S2. The circulation direction with regular and reversed connection lines during RCA‐CRRT is shown in Figures S3 and S4.

### 2.4 Data collection

We collected the following demographic and clinical information during CRRT treatment: mode of connection (regular or reversed), age, sex, femoral vein site, blood flow, citrate flow, and primary diagnosis. Blood sampling from the CVC line and that from the circuit line was synchronously performed by two experienced researchers. Acid‐base and electrolyte parameters were determined using the COBAS b 123 system (Roche Diagnostics, Basel, Switzerland). Potential adverse events such as bleeding, citrate accumulation, hypocalcaemia, hypotension, and catheter‐related complications were recorded in each CRRT circuit.

### 2.5 Statistical analysis

IBM SPSS Statistics for Windows, version 19 (IBM Corp., Armonk, New York) was used for all statistical analyses. Normally distributed data were expressed as mean ± SD. Categorical variables were presented as absolute frequencies. The paired samples *t* test was performed to compare continuous numerical variables with a normal distribution. Statistical significance was set at a two‐tailed *P* value <.05.

## 3 RESULTS

### 3.1 Patient characteristics

From December 2019 to June 2020, 60 adult patients with 128 CRRT circuits who underwent the RCA‐CVVHDF protocol via femoral vein access were included. Among these, 98 (76.6%) circuits had regular connections, while 30 (23.4%) circuits had r reversed connections. The baseline characteristics of the study participants are summarized in Table 1.

**TABLE 1:** Baseline characteristics of the study participants

Characteristics	Value
Filter count (n)	128
Connection type, n (%)	
Regular connection	98 (76.6)
Reversed connection	30 (23.4)
Sex, n (%)	
Male	35 (58.3)
Women	25 (41.7)
Age, mean (SD), y	55.8 ± 13.35
Weight, mean (SD), kg	66.3 (5.11)
Vascular access, n (%)	
Right femoral vein	34 (56.7)
Left femoral vein	26 (43.3)
Citrate dose, mean (SD), mmol/L	2.62 (1.88)
APACHE II, mean (SD)	26.38 (7.98)
Primary disease diagnosis (%)	
Sepsis	18 (30.0)
Severe pancreatitis	16 (26.7)
Severe pneumonia	15 (25.0)
Others	11 (18.3)

### 3.2 Acid‐base and electrolyte parameters in the CVC line and in the circuit line

**FIGURE 1:**
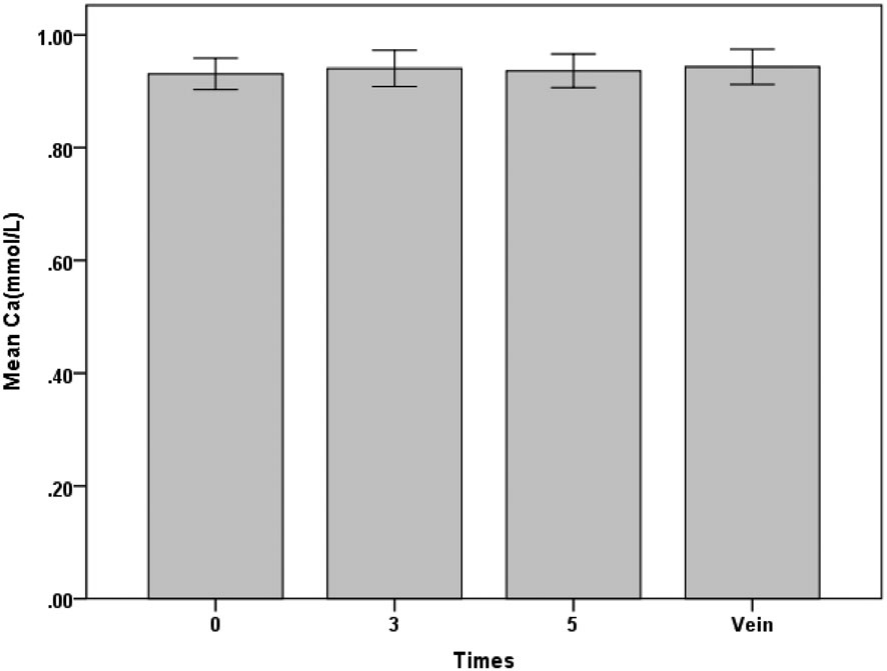
Error histogram of ionized calcium under the regular connection

**FIGURE 2:**
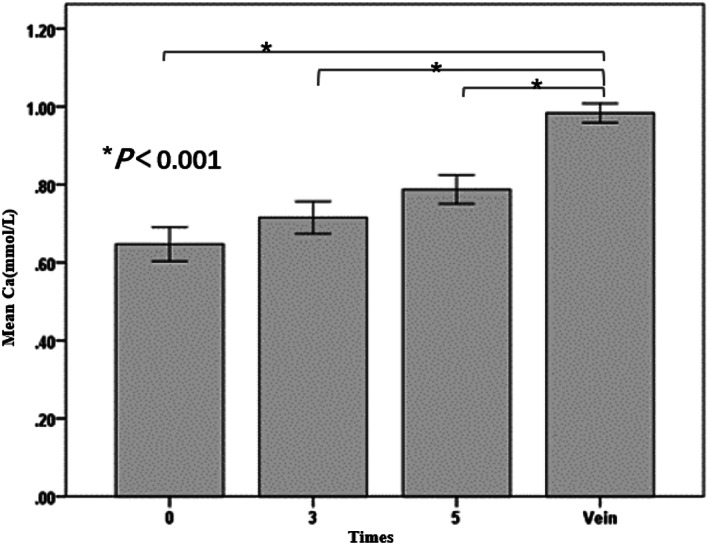
Error histogram of ionized calcium under the reversed connection

Among the circuits with regular connection between the catheter and the CRRT circuit, no statistically significant differences were observed in any of the acid‐base or electrolyte parameters between samples from the CVC line and those from the circuit line (*P* > .05) (Table 2 and Figure 1). No differences were observed at all three time points between the ionized calcium levels in the circuit line and those in the CVC line (0.93 ± 0.07, 0.94 ± 0.08, and 0.94 ± 0.08 vs 0.93 ± 0.07, *P* > .05). Among the circuits with reversed connection (Table 2 and Figure 2), the pre‐filter ionized calcium concentrations were 0.98 ± 0.07 mmol/L in the CVC line and 0.65 ± 0.12 mmol/L, 0.72 ± 0.11 mmol/L, and 0.78 ± 0.99 mmol/L in the circuit lines at 0, 3, and 5 minutes, respectively. Ionized calcium levels in the circuit line were dramatically decreased at all three time points compared with the levels in the CVC line (*P* < .001), whereas no change was found in terms of other acid‐base or electrolyte parameters (*P* > .05).

**TABLE 2:** Comparison of acid‐base and electrolyte parameters between the central venous catheter (CVC) line and circuit lines

Acid‐base and electrolyte parameters	Regular connection (n = 98)				Reversed connection (n = 30)			
	CVC line	Circuit line (0 min)	Circuit line (3 min)	Circuit line (5 min)	Venous line	Circuit line (0 min)	Circuit line (3 min)	Circuit line (5 min)
PH	7.35 ± 0.07	7.35 ± 0.05	7.35 ± 0.06	7.35 ± 0.06	7.33 ± 0.08	7.33 ± 0.08	7.33 ± 0.08	7.33 ± 0.08
cHCO_3_^−^ (mmol/L)	25.14 ± 2.31	25.69 ± 1.93	25.63 ± 1.92	25.87 ± 1.77	24.86 ± 2.67	24.61 ± 3.47	24.95 ± 3.00	25.46 ± 2.80
Hct (%)	26.71 ± 4.73	25.98 ± 5.03	26.30 ± 4.71	26.10 ± 4.74	29.65 ± 4.06	29.43 ± 4.17	29.26 ± 4.04	29.08 ± 3.74
Glu (mmol/L)	10.62 ± 3.16	10.30 ± 2.96	9.82 ± 2.89	9.76 ± 3.04	8.45 ± 2.12	8.48 ± 1.90	8.16 ± 1.63	8.45 ± 2.12
Lac (mmol/L)	1.94 ± 0.67	1.83 ± 0.50	1.76 ± 0.49	1.81 ± 0.50	2.79 ± 2.04	2.65 ± 1.92	2.80 ± 1.99	2.77 ± 1.95
K^+^ (mmol/L)	4.29 ± 0.46	4.25 ± 0.43	4.25 ± 0.44	4.28 ± 0.45	3.97 ± 0.40	3.85 ± 0.43	4.03 ± 0.48	3.92 ± 043
Na^+^ (mmol/L)	135.43 ± 3.72	135.77 ± 2.94	135.50 ± 3.38	135.55 ± 3.14	135.76 ± 2.75	135.84 ± 2.68	136.01 ± 3.08	136.45 ± 3.12
iCa^2+^ (mmol/L)	0.93 ± 0.07	0.93 ± 0.07	0.94 ± 0.08	0.94 ± 0.08	0.98 ± 0.07	0.65 ± 0.12*	0.72 ± 0.11*	0.78 ± 0.99*

*Note*: Compared with the acid‐base and electrolyte parameters in the vein, *P < .001, the others, *P* > .05.

Abbreviations: BE, base excess; Glu, glucose; Hb, haemoglobin; HCO_3_^−^, standard bicarbonate; HCT, haematocrit; Lac, lactate; PCO_2_, partial pressure of carbon dioxide; PO_2_, partial pressure of oxygen.

### 3.3 Adverse events

Hypotension was the only adverse event observed in the present study. Altogether, 12 (20%) patients developed new‐onset hypotension (a decrease in the systolic blood pressure ≥20 mm Hg or a decrease in the mean arterial pressure ≥10 mm Hg^20^) within 1 hour of CRRT initiation, which returned to baseline after 2 hours of CRRT treatment. Among patients who had hypotension, nine patients had a regular connection, and the remaining three had a reversed connection. No episodes of bleeding or citrate accumulation were observed during CVVHDF. None of the patients developed hypocalcaemia. None of the patients with regular connections had catheter‐related complications during the first 3 days. The patients in the reversed‐connection group also attained sufficient blood flow rates during the first 3 days. None of the patients had extracorporeal circuit clotting due to blood sampling.

## 4 DISCUSSION

In this prospective cohort study, we collected blood samples to monitor acid‐base and electrolyte parameters and their levels were compared between samples from the circuit lines (at 0, 3, and 5 minutes) as controls and those from the CVC line (at 0 minute) during the second hour after CRRT initiation. We observed that in cases with regular connections, the acid‐base and electrolyte parameters could be reliably monitored through the circuit line during CRRT with RCA while maintaining the normal functioning of the system. However, in cases with reversed connections, it was inaccurate to monitor the systemic ionized calcium concentration through the arterial sampling port in the circuit line during RCA‐CRRT despite the CRRT downtime (3 minutes or 5 minutes).

Citrate acts as an anticoagulant by chelating the ionized calcium in the extracorporeal circuit and blocking the coagulation.^6^ The presence of citrate in the circuit line during CRRT treatment could affect the accuracy of determining the levels of acid‐base and electrolyte parameters sampled from the circuit line. Since citrate concentration is associated with access recirculation, our study also set 3 minutes and 5 minutes as controls. During this period, CRRT switched to the replacement state with only the blood pump running to decrease the citrate concentration of the circuit line. Previous studies have reported that access recirculation rate was below 5% with a correct connection, but increased to 13% with a reversed connection.^13^, ^14^, ^21^, ^22^ Increased access recirculation due to reversed lines could impair dialysis adequacy and test accuracy of blood samples from the circuit lines. Citrate might chelate more calcium due to increased access recirculation in reversed lines, leading to a lower calcium concentration in the blood sampled from the circuit line than that from the CVC line.

Access recirculation might explain the discrepancy observed in the present study between the ionized calcium levels in the CVC line and those in the circuit line in reversed connections. Consistent with the results of the present study, two case reports using blood sampling from the circuit line during CRRT with RCA also showed an inevitable influence of vascular access recirculation on ionized calcium levels.^18^, ^19^ Recirculation could lead to low calcium levels in the blood circulating in the circuit. Thus, we do not recommend reversing the connection during CRRT while using RCA as an anticoagulant. Moreover, caregivers should be extremely careful in modifying the setting of the RCA protocol when calcium levels are measured from the circuit line.^19^

In clinical practice, it is critical to monitor the acid‐base and electrolyte parameters during CRRT.^23^ Nursing time is mostly devoted to blood sampling and essential parameter monitoring during CRRT with RCA.^24^ Reversing the catheter connection is frequently performed to decrease the input pressures for various reasons. However, the accuracy of the acid‐base and electrolyte parameters in clinical practice with a reversed connection mode should be noted. Blood sampling from patients' arterial lines has been suggested instead of sampling from the circuit line to avoid the effects of vascular access recirculation on the determination of ionized calcium.^25^ We suggest that blood can be sampled from the circuit line in a regular connection and from patients' arterial or venous lines in a reversed connection during CRRT with RCA.

### 4.1 Limitations

Our study has several limitations. This study is a cohort study with a small sample size limited to a single centre. This could weaken the quality of evidence to some extent. Nevertheless, we designed this prospective study based on the best interests of patients. We included only the patients with femoral vein access, because it is the most commonly used vein for CRRT catheter in our department due to ease of operation and convenience in clinical practice. Since we did not measure the access recirculation rate, the relationship between access recirculation rate and calcium concentration requires further analysis.

## 5 IMPLICATIONS FOR PRACTICE

Reversing the connection mode of dysfunctional dual‐lumen CVCs is frequently performed to attain sufficient blood flow rates in clinical practice. The inaccuracy of the ionized calcium concentration in blood samples collected from a reversed connection mode should receive due consideration. We suggest sampling from an arterial or venous line rather than from the circuit line while using a reversed connection during CRRT with RCA.

## 6 CONCLUSIONS

The present study showed the high reliability of monitoring acid‐base and electrolyte parameters through the circuit line during CRRT with RCA in case of a regular connection. However, in case of a reversed connection, pre‐filter ionized calcium concentrations determined through the circuit lines could be lower than those determined through traditional venous lines. We suggest performing blood sampling from arterial or venous lines of patients rather than from the circuit lines in a reversed connection during CRRT operation. Future studies are needed to confirm the findings of the present study, particularly in different CRRT protocols involving different anticoagulants and catheter types.

## AUTHOR CONTRIBUTIONS

All authors contributed to the design and implementation of the research, to the analysis of the results, and to the writing of the manuscript.

## Supporting information


**Figure S1.** The progress of circuits through the study.
**Figure S2.** Circuit with sampling point (CVVHDF).
**Figure S3.** The circulation direction with regular‐connected lines during RCA‐CRRT.
**Figure S4.** The circulation direction with reversed‐connected lines during RCA‐CRRT.

## References

[1] Ronco C , Ricci Z , De Backer D , et al. Renal replacement therapy in acute kidney injury: controversy and consensus. Crit Care. 2015;19:146. 25887923 10.1186/s13054-015-0850-8 PMC4386097

[2] Stucker F , Ponte B , Tataw J , et al. Efficacy and safety of citrate‐based anticoagulation compared to heparin in patients with acute kidney injury requiring continuous renal replacement therapy: a randomized controlled trial. Crit Care. 2015;19:91. 25881975 10.1186/s13054-015-0822-z PMC4364313

[3] Schilder L , Nurmohamed SA , Bosch FH , et al. Citrate anticoagulation versus systemic heparinisation in continuous venovenous hemofiltration in critically ill patients with acute kidney injury: a multi‐center randomized clinical trial. Crit Care. 2014;18:472. 25128022 10.1186/s13054-014-0472-6 PMC4161888

[4] Zarbock A , Kullmar M , Kindgen‐Milles D , et al. Effect of regional citrate anticoagulation vs systemic heparin anticoagulation during continuous kidney replacement therapy on dialysis filter life span and mortality among critically ill patients with acute kidney injury: a randomized clinical trial. JAMA. 2020;324:1629‐1639. 33095849 10.1001/jama.2020.18618 PMC7585036

[5] KDIGO. Kidney Disease: Improving Global Outcomes (KDIGO) Acute Kidney Injury Work Group . KDIGO clinical practice guideline for acute kidney injury. Kidney Int. 2012;2:1‐138.

[6] Oudemans‐van Straaten HM , Ostermann M . Bench‐to‐bedside review: citrate for continuous renal replacement therapy, from science to practice. Crit Care. 2012;16:249. 23216871 10.1186/cc11645 PMC3672558

[7] Palevsky PM , Liu KD , Brophy PD , et al. KDOQI US commentary on the 2012 KDIGO clinical practice guideline for acute kidney injury. Am J Kidney Dis. 2013;61:649‐672. 23499048 10.1053/j.ajkd.2013.02.349

[8] Zhang L , Liao Y , Xiang J , et al. Simplified regional citrate anticoagulation using a calcium‐containing replacement solution for continuous venovenous hemofiltration. J Artif Organs. 2013;16:185‐192. 23271571 10.1007/s10047-012-0680-2

[9] Fall P , Szerlip HM . Continuous renal replacement therapy: cause and treatment of electrolyte complications. Semin Dial. 2010;23:581‐585. 21166876 10.1111/j.1525-139X.2010.00790.x

[10] Akhoundi A , Singh B , Vela M , et al. Incidence of adverse events during continuous renal replacement therapy. Blood Purif. 2015;39:333‐339. 26022612 10.1159/000380903

[11] Yessayan L , Yee J , Frinak S , Szamosfalvi B . Continuous renal replacement therapy for the management of acid‐base and electrolyte imbalances in acute kidney injury. Adv Chronic Kidney Dis. 2016;23:203‐210. 27113697 10.1053/j.ackd.2016.02.005

[12] Niyyar VD , Chan MR . Interventional nephrology: catheter dysfunction–prevention and troubleshooting. Clin J Am Soc Nephrol. 2013;8:1234‐1243. 23824200 10.2215/CJN.00960113

[13] Atapour A , Mosakazemi M , Mortazavi M , Beigi A , Shahidi S . Access recirculation in jugular venous catheter in regular and reversed lines. Iran J Kidney Dis. 2008;2:91‐94. 19377215

[14] Hassan HA , Frenchie DL , Bastani B . Effect of reversal of catheter ports on recirculation: comparison of the PermCath with Tesio Twin Catheter. ASAIO J. 2002;48:316‐319. 12059008 10.1097/00002480-200205000-00019

[15] Carson RC , Kiaii M , MacRae JM . Urea clearance in dysfunctional catheters is improved by reversing the line position despite increased access recirculation. Am J Kidney Dis. 2005;45:883‐890. 15861354 10.1053/j.ajkd.2005.01.029

[16] Level C , Lasseur C , Chauveau P , et al. Performance of twin central venous catheters: influence of the inversion of inlet and outlet on recirculation. Blood Purif. 2002;20:182‐188. 11818683 10.1159/000047007

[17] Pannu N , Jhangri GS , Tonelli M . Optimizing dialysis delivery in tunneled dialysis catheters. ASAIO J. 2006;52:157‐162. 16557101 10.1097/01.mat.0000202081.13974.39

[18] Degraeve A , Danse E , Laterre PF , Hantson P , Werion A . Regional citrate anticoagulation and influence of recirculation on ionized calcium levels in the circuit. J Artif Organs. 2019;22:341‐344. 31444670 10.1007/s10047-019-01125-3

[19] Chenouard A , Liet J‐M . Regional citrate anticoagulation: beware of recirculation phenomenon. Ther Apher Dial. 2017;21:206‐207. 28195402 10.1111/1744-9987.12505

[20] Workgroup KD . K/DOQI clinical practice guidelines for cardiovascular disease in dialysis patients. Am J Kidney Dis. 2005;45:S1‐S153. 15806502

[21] Senecal L , Saint‐Sauveur E , Leblanc M . Blood flow and recirculation rates in tunneled hemodialysis catheters. ASAIO J. 2004;50:94‐97. 14763498 10.1097/01.mat.0000104825.33101.7c

[22] Kousoula V , Georgianos PI , Mavromatidis K , et al. Reversed connection of cuffed, tunneled, dual‐lumen catheters with increased blood flow rate maintains the adequacy of delivered dialysis despite the higher access recirculation. Int Urol Nephrol. 2019;51:1841‐1847. 31471844 10.1007/s11255-019-02268-1

[23] Daugirdas JT , Depner TA , Inrig J , et al. KDOQI clinical practice guideline for hemodialysis adequacy: 2015 update. Am J Kidney Dis. 2015;66:884‐930. 26498416 10.1053/j.ajkd.2015.07.015

[24] Houlle‐Veyssiere M , Courtin A , Zeroual N , Gaudard P , Colson PH . Continuous venovenous renal replacement therapy in critically ill patients: a work load analysis. Intensive Crit Care Nurs. 2016;36:35‐41. 27283118 10.1016/j.iccn.2016.04.001

[25] Morabito S , Pistolesi V , Tritapepe L , Fiaccadori E . Regional citrate anticoagulation for RRTs in critically ill patients with AKI. Clin J Am Soc Nephrol. 2014;9:2173‐2188. 24993448 10.2215/CJN.01280214 PMC4255392

