# Supplementary material for: Reliability of monitoring acid‐base and electrolyte parameters through circuit lines during regional citrate anticoagulation‐continuous renal replacement therapy
Source: Nurs Crit Care. 2021 Aug 11;27(5):646–51. doi: 10.1111/nicc.12696 (PMC9540182; doi:10.1111/nicc.12696)
Supplement: Supplementary file 1 — Figure S1. The progress of circuits through the study. Figure S2. Circuit with sampling point (CVVHDF). Figure S3. The circulation direction with regular‐connected lines during RCA‐CRRT. Figure S4. The circulation direction with reversed‐connected lines during RCA‐CRRT. [file NICC-27-646-s001.docx]

**Figure Legends**


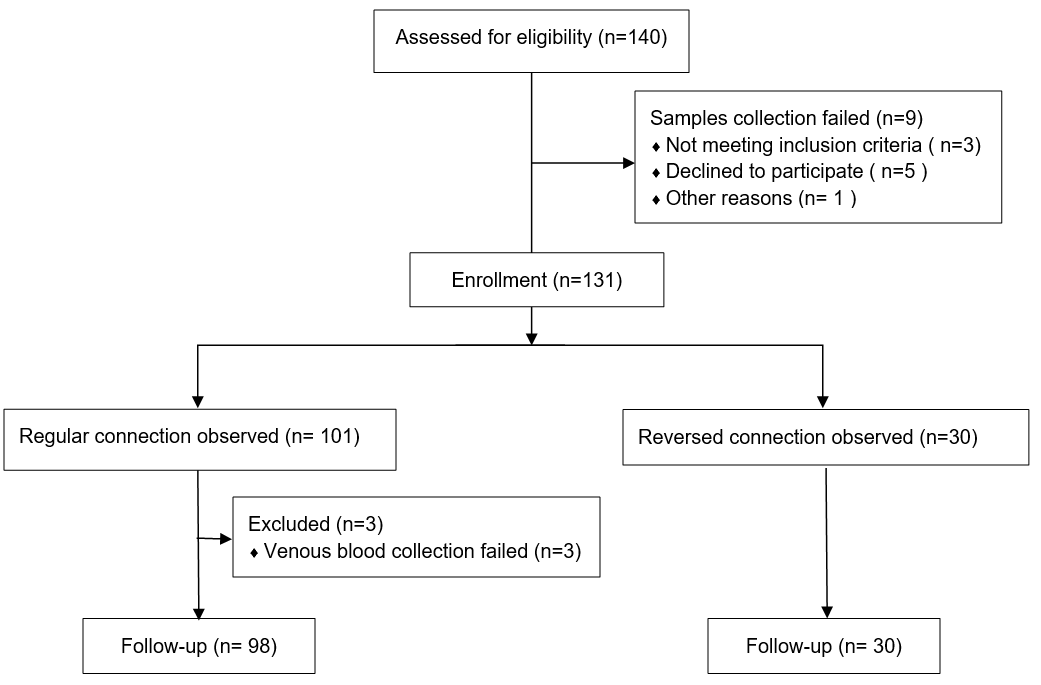


**Supplementary Fig. 1** **The progress of circuits** **through the study**


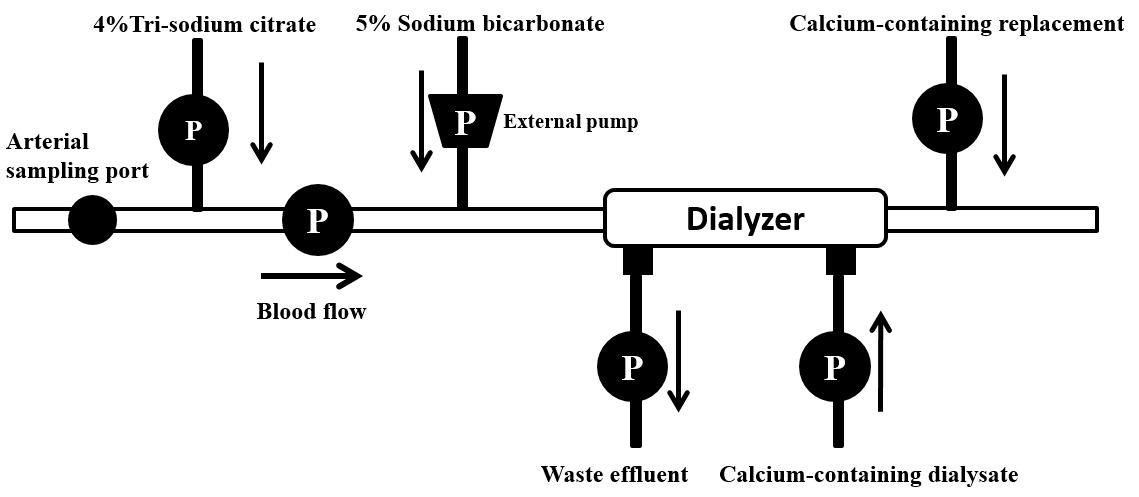


**Supplementary Fig. 2. Circuit with sampling point (CVVHDF)**


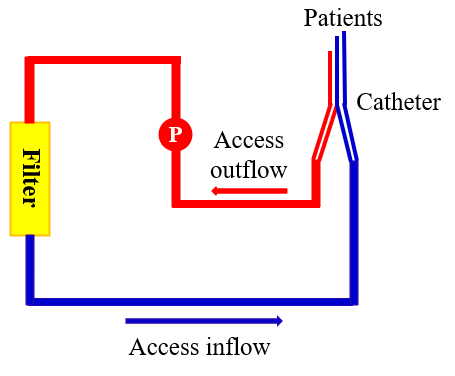

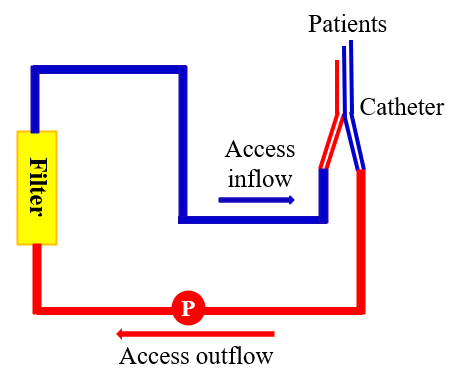


**Supplementary** F**ig.3**

**The circulation direction with regular-connected lines during RCA-CRRT**

**Supplementary Fig.4**

**The circulation direction with reversed-connected lines during RCA-CRRT**
